# An Improved Direction Finding Algorithm Based on Toeplitz Approximation

**DOI:** 10.3390/s130100746

**Published:** 2013-01-07

**Authors:** Qing Wang, Hua Chen, Guohuang Zhao, Bin Chen, Pichao Wang

**Affiliations:** School of Electronic and Information Engineering, Tianjin University, Tianjin 300072, China; E-Mails: wqelaine@tju.edu.cn (Q.W.); keyi1110@163.com (G.Z.); chenbintju@tju.edu.cn (B.C.); wangpichao@tju.edu.cn (P.W.)

**Keywords:** DOA estimation, fourth order cumulants, MUSIC-like, Toeplitz approximation, spatially-white noise, spatially-color noise

## Abstract

In this paper, a novel direction of arrival (DOA) estimation algorithm called the Toeplitz fourth order cumulants multiple signal classification method (TFOC-MUSIC) algorithm is proposed through combining a fast MUSIC-like algorithm termed the modified fourth order cumulants MUSIC (MFOC-MUSIC) algorithm and Toeplitz approximation. In the proposed algorithm, the redundant information in the cumulants is removed. Besides, the computational complexity is reduced due to the decreased dimension of the fourth-order cumulants matrix, which is equal to the number of the virtual array elements. That is, the effective array aperture of a physical array remains unchanged. However, due to finite sampling snapshots, there exists an estimation error of the reduced-rank FOC matrix and thus the capacity of DOA estimation degrades. In order to improve the estimation performance, Toeplitz approximation is introduced to recover the Toeplitz structure of the reduced-dimension FOC matrix just like the ideal one which has the Toeplitz structure possessing optimal estimated results. The theoretical formulas of the proposed algorithm are derived, and the simulations results are presented. From the simulations, in comparison with the MFOC-MUSIC algorithm, it is concluded that the TFOC-MUSIC algorithm yields an excellent performance in both spatially-white noise and in spatially-color noise environments.

## 1. Introduction

During the last few decades, DOA estimation, which has been widely applied in the fields of sonar, radar, wireless communication, aeronautics, *etc.* [1], plays a significant role in array signal processing. Among various DOA estimation methods, the so called subspace approaches based upon the eigende composition of the sample covariance matrix possess very appealing features for narrowband case. The multiple signal classification (MUSIC) [2] algorithm which pertains to the aforementioned subspace method can achieve favorable resolution when compared with conventional algorithms, for instance, Capon beam forming algorithm. However, MUSIC and its improved versions require the prior knowledge of the noise characteristics of the sensors [3]. Moreover, the total number of signals impinging on the array must be less than the number of sensors [4]. Therefore, these problems limit the applicability of the MUSIC algorithms to practical environments.

Conventional array processing techniques utilize only the second order statistics (SOSs) of the received signal, which may have suboptimal performance due to the transmitted signals, combining with additive Gaussian noise, are often non-Gaussian in real applications, e.g., as in a communications system [5]. In addition, the SOSs have the drawback of being sensitive to the type of the noise. Fortunately, the fourth order cumulants (FOC) have been shown to be promising in solving these problems, since it is possible to recover phase information with cumulants for non-Gaussian processes. Furthermore, the FOC are asymptotically blind to the Gaussian process. Thus, it is not necessary to model or estimate the noise covariance, as long as the noise is Gaussian, which is a rational assumption in many practical situations. Because of these advantages, we can substitute FOC for SOC with MUSIC algorithms. The method proposed in [6] is defined as the MUSIC-like algorithm based on FOC, which is also called fourth order cumulants MUSIC (FOC-MUSIC) algorithm in this paper. With FOC, the effective array aperture of a physical array can be extended, which makes the number of estimated signals greater than or equal to that of sensors possible.

But the conventional MUSIC-like algorithms have high computational requirements as a result of the great number of redundant information contained in the FOC matrix as well as the rigorous requirements of sampling snapshots for the FOC matrix estimation. To mitigate these drawbacks, a fast MUSIC-like algorithm (the MFOC-MUSIC algorithm) is proposed to reduce the computational complexity effectively [7]. On the other hand, the FOC matrix infinitely approaches the theoretical value when the number of the snapshots goes to infinity [8]. However, because of the existence of the estimation error of the FOC matrix, the performance of the MFOC-MUSIC algorithm cannot be asymptotically optimal. So, the proposed algorithm in [9] successfully applies the Toeplitz approximate method to the cumulants domain, which mainly focuses on the amplitude and phase error model. In this paper, the MFOC-MUSIC algorithm in conjunction with Toeplitz approximation, which is termed the TFOC-MUSIC algorithm, is proposed. The emphasis of the paper is on the investigation of how the algorithm is impacted by sampling snapshots. Firstly, in the TFOC-MUSIC algorithm, the reduced-rank FOC matrix is obtained by removing the redundant information encompassed in the primary FOC matrix. Meanwhile, the effective aperture of the virtual array remains unchanged. Then, with applying the Toeplitz approximation, the Toeplitz structure of the reduced-rank FOC matrix is recovered. And finally, by using the MUSIC algorithm, the direction of arrival signals can be estimated.

The rest of this paper is organized as follows. Section 2 introduces the system model and the MUSIC-like algorithm. In Section 3, the TFOC-MUSIC algorithm is described in detail. Section 4 presents comparative simulation results that show the effectiveness of the proposed algorithm. Finally, we conclude this paper in Section 5.

Throughout the paper, lower-case boldface italic letters denote vectors, upper-case boldface italic letters represent matrices, and lower and upper-case italic letters stand for scalars. The symbol * is used for conjugation operation, and the notations (*x*)*^T^* and (*x*)*^H^* represent transpose and conjugate transpose, respectively. We use *E*(*x*), *cum*(*x*) and ⊗ to indicate the expectation operator, the cumulants, and the Kronecker product, separately.

## 2. System Model and the MUSIC-Like Algorithm

### 2.1. System Model

Assume that *M* far-field narrowband plane wave signals *s_l_*(*t*), (*l* = *1*, …, *M*) impinging on a uniform linear array (ULA) of *N* identical omni-directional sensors with *λ/2* inter-element spacing, where *λ* is the wavelength of the carrier. We suppose that the source signals are stationary and mutually independent, and that the noises with variance *σ^2^* are statistically independent to the signals as well.

Then, the signal received in time *t* at the *i*th sensor can be expressed as

(1) $$\begin{array}{cc} x_{i}\;(t)=\sum_{l=1}^{M}S_{l}\;(t)a_{i}\;(\theta_{l})+n_{i}\;(t), & i=1,\ldots \ldots ,N \end{array}$$

where *α_i_*(*θ_i_*) is the spacial response of *i*th sensor corresponding to the *l*th source

(2) $$a_{i}\;(\theta_{l})=\exp(j\pi i\sin\theta_{l})$$

In matrix form, it becomes *a*(*θ_l_*) = [*a_1_*(*θl*), …, *a_N_*(*θ_l_*)]*^T^*.

Then, rewriting Equation (1) in matrix form, we obtain

(3) X(t)=AS(t)+N(t)

where ***X***(*t*) = [*x_1_*(*t*), …, *x_N_*(*t*)]*^T^* is the *N* × 1 received signal vector, ***S***(*t*) = [*s_1_*(*t*), …, *s_M_*(*t*)]*^T^* is the *M* × 1 transmitted signal vector, ***A*** = [*a*(*θ_l_*), …, *a*(*θ_M_*)] is the *N* × *M* steering matrix defined as array manifold and ***N***(*t*) = [*n_1_*(*t*), …, *n_N_*(*t*)]*^T^* represents the *N* × 1 complex Gaussian noise vector.

The MUSIC algorithm makes use of the covariance matrix of the data received by the sensor array, denoted by

(4) $$\begin{array}{cc} R_{2} & =E[X(t)X^{H}(t)]\\ & =AR_{s}A^{H}+\sigma^{2}I \end{array}$$

where ***R****_S_* denotes the covariance matrix of radiating signals, and ***I*** is the *N* × *N* identity matrix. The eigende composition is based on ***R****_2_*, and then the signal and noise subspaces can be achieved, respectively.

### 2.2. The MUSIC-LIKE Algorithm

For symmetrically distributed signals, their odd-order cumulants are usually zero. Therefore, even-order cumulants are the main objects of investigation, in particular with the FOC. There exist various definitions about the FOC matrix. For zero mean stationary random process, the *4*th order cumulants can be defined as [6]

(5) $$\begin{array}{ll} \text{cum}\;(k_{1},k_{2},k_{3}^{*},k_{4}^{*})= & E(x_{k_{1}}\;(t)x_{k_{2}}\;(t)x_{k_{3}}^{*}\;(t)x_{k_{4}}^{*}\;(t))-\\ & E(x_{k_{1}}\;(t)x_{k_{3}}^{*}\;(t))E(x_{k_{2}}\;(t)x_{k_{4}}^{*}\;(t))-\\ & E(x_{k_{1}}\;(t)x_{k_{4}}^{*}\;(t))E(x_{k_{2}}\;(t)x_{k_{3}}^{*}\;(t))-\\ & E[x_{k_{1}}\;(t)x_{k_{2}}\;(t)]E[x_{k_{3}}^{*}\;(t)x_{k_{4}}^{*}\;(t)]-\\ & k_{1},k_{2},k_{3},k_{4}\in [1,N] \end{array}$$

where *x_km_* (m = 1, 2, 3, 4) is the stochastic process. For simplicity, Equation (5) can be collected in matrix form, denoted by cumulants matrix ***C****_4_*, and *cum*(*k_1_*, *k_2_*, k_3_*, k_4_*) appears as the [(*k_1_* − 1)*N* + *k_2_*]th row and [(*k_3_* − 1)*N* + *k_4_*]th column of ***C****_4_*.

The *2q*th order data statistics are arranged generally controls the geometry and the number of Virtual Sensors (VSs) of the Virtual Array (VA) and, thus, the number of sources that can be processed by a *2q*th order method exploiting the algebraic structure of *2q*th order circular cumulants matrix ***C****_2q,x_*. Introduce *g* as an arbitrary integer (*0* ≤ *g* ≤ *q*), for different arrangement of ***C****_2q,x_*(*g*). To optimize the maximum number of VSs with respect to *g*, the optimal arrangement of the data statistics was solved in [10] that *g_opt_* = *q/*2 if *q* is even, and *g_opt_* = (*q* + 1)/2 if *q* is odd. But In the particular case of a ULA of *N* identical sensors, it has been shown that all the considered arrangements of the data statistics are equivalent and give rise to VA with $N_{2q}^{g}=q(N-1)+1\text{VSs}$. Whereas for UCA, results differs, which was not discussed in this paper. If source signal is independent of each other, ***C****_4_* can be written as Equation (6), which corresponds to the ***C****_2q,x_*(*g*) matrix for the situation of *q* = 2 and *g* = 2 in [10,11].

(6) $$C_{4}[(k_{1}-1)N+k_{2},(k_{3}-1)N+k_{4}]=BC_{s}B^{H}$$

(7) $$C_{s}=\text{diag}(\text{cum}(s_{1}\;(t),s_{1}\;(t),s_{1}^{*}\;(t)s_{1}^{*}\;(t)),\ldots \ldots ,\text{cum}\;(s_{M}\;(t),s_{M}\;(t),s_{M}^{*}\;(t),s_{M}^{*}\;(t)))$$

where ***B*** and ***C****_s_* indicate the extended array manifold and the FOC matrix of radiating signals, respectively. Although this is suboptimal [10], it can also be able to process up to $N_{2q}^{g}-1=q(N-1)$ non-Gaussian sources. As in the case of the MUSIC algorithm, we can compute the eigendecomposition of ***C****_4_*. Its eigenvectors (*e_1_*, ……, *e_N_*_2_) are separated into the signal and noise subspaces according to the descending order of the eigenvalues (*λ_1_*, ……, *λ_N_*_2_). The signal subspaces ***E****_S_* spanned by (*e_1_*, ……, *e_M_*) is identical to ***B*** = (***b***(*θ_1_*), ……, ***b***(*θ_M_*)), where ***b***(*θ_1_*) = ***a***(*θ_1_*)***a***(*θ_1_*). The spaces spanned by (*e_M_*_+1_, ……, *e_N_*_2_) is called the noise subspaces ***E****_N_* that is perpendicular to ***B***. DOAs are acquired by exploiting the orthogonality that ***B****^H^****E****_N_* = **0** like the MUSIC algorithm. Then, in the MUSIC-like algorithm, the spatial spectrum *ϕ*(*θ*) is defined as:

(8) $$\phi (\theta )=\frac{1}{b{\left(\theta \right)}^{H}E_{N}{E_{N}}^{H}b(\theta )}$$

(9) b(θ)=a(θ)⊗a(θ)

where *θ* ∈ [−90°, 90°]. According to the property of the Kronecker product, it is obvious that ***b***(*θ*) is a *N^2^* × 1 vector, which means that the array aperture of ULA is extended and allows signals to be estimated no less than sensors. The *M* source directions can be obtained by searching the peaks of *P*(*θ*) with *θ* confined to [−90°, 90°].

## 3. The Proposed Algorithm

### 3.1. The Effective Array Aperture Extended

As proven in [12], for the MUSIC-like algorithm, an array of arbitrary identical physical sensors can be extended to a maximum of *N^2^* − *N* + 1 virtual ones. Specifically, the number of virtual sensors is showed in [12] to be 2*N* − 1 with regard to ULA. In order to analyze the array effective aperture of ULA, we assume that there exist three real sensors, namely *N* = 3 in space and specialize Equation (9) as follows

(10) $$b(\theta )={\left[1,p,p^{2},p,p^{2},p^{3},p^{2},p^{3},p^{4}\right]}^{T}$$

where *p* = *exp*(*jπsinθ*), while ***a***(*θ*) can be expressed as

(11) $$a(\theta )={\left[1,p,p^{2}\right]}^{T}$$

Comparing Equation (10) with (11), we can see that only two items are different implying that the effective array aperture is extended with 2*N* − 1 = 5 elements, and the remaining items of Equation (10) are redundant. With the increase of the sensors' number, ***b***(*θ*) is highly redundant which leads to a heavy computational burden. Next, we will investigate the redundant elements in ***b***(*θ*), and describe how to remove redundancy and to improve the computational efficiency.

The effective number of different sensors $N_{2q}^{g}$ is smaller than the upper-bound *N_max_*[*2q, g*] for ULA [10], which means redundancy in the virtual array can be removed using the reduced-dimension method. But for UCA, $N_{2q}^{g}$ is equal to *N_max_* [*2q*, *g*] in the *4*th order array processing method. *i.e.*, *N_4_^2^* = *N_max_*[*4*, *2*] = *N(N* + 1)/2, for *N* is odd [10]. So the proposed algorithm cannot reduce the computational complexity for UCA in reduced-dimension method.

### 3.2. The TFOC-MUSIC Algorithm

In this section, we describe the MFOC-MUSIC algorithm combined with Toeplitz approximation in detail. To begin with, the MFOC-MUSIC algorithm is described. From Equation (10), we know that there is a lot of redundancy in expanded steering vector ***b***(*θ*). In general, for the *N*-array ULA, only from 1 to *N* and all *kN*(*k* = 2, …, *N*) items of the expanded steering vector ***b***(*θ*) are valid for the MUSIC-like algorithm, while others are redundant items. Owing to the steering vector of each element in accordance with the corresponding element, accordingly, ***C****_4_* definitely exists a large number of duplicate values. The MFOC-MUSIC algorithm is to remove the redundant information, at the same time, to extend the array aperture.

In light of the above analysis, it can be seen that ***C****_4_* has *2N* − 1 different elements, that is, the rows and columns number of ***C****_4_* is 2*N* − 1. Next, we define a (2*N* − 1) × (2*N* − 1) dimension matrix denoted by ***R****_4_*. Now let's take out the *1*th to *N*th and all *kN*th (*k* = 2, …, *N*) rows of ***C****_4_* in sequence, and then store these rows in the *1*th to (2*N* − 1)th row of the newly defined matrix ***R****_4_*. The same operation is performed on the 1th to *N*th and all *kN*th (*k* = 2, …, *N*) columns of ***C****_4_* to obtain the *1*th to (2*N* − 1)th columns of ***R****_4_*.

Like in Equation (6), ***R****_4_* has a similar mathematical expression as follows

(12) $$R_{4}=DC_{s}D^{H}$$

where ***D*** designates the extended array manifold without redundancy, and each column of ***D*** has the form of [1, …, *p^2N^*^−^*^2^*]*^T^* recording ***d***(*θ*). Here, we obtain reduced-dimension ***R****_4_* including the all information of the extended array without redundancy, which ensures that the amount of calculation of the MFOC-MUSIC algorithm is greatly reduced when compared to the MUSIC-like algorithm.

In practical applications, we do not have access to true ***C****_4_*. Instead, we utilize the estimated ***R̂***_4_ in lieu of ***C****_4_* from the received data by array measurements, subsequently, ***Ĉ***_4_ which signifies the estimation value of ***R****_4_* is in place of ***R****_4_*, too. In order to obtain satisfactory results, a large number of sampling snapshots are required for cumulants domain processing. As is well known that the signal covariance matrix ***R****_2_* of an ideal ULA is Toeplitz [13], so do ***R****_4_*. However, in the case of finite snapshots, the above desired properties cannot be preserved. To recover the Toeplitz property of ***R̂***_4_, the Toeplitz approximation, which was primarily presented for DOA estimation of coherent sources [14], is employed to generate a Toeplitz matrix ***R̂***_4_*_T_* from the biased matrix ***R̂***_4_. It is shown that the eigenstructure of ***R̂***_4_*_T_* infinitely approaches that of ***R****_4_*, as sampling snapshots gradually increase. And then the TFOC-MUSIC algorithm takes advantage of ***R̂***_4_*_T_* for eigendecomposition rather than ***R̂***_4_ to get the signal and noise subspaces representing ***Û****_S_* and ***Û****_N_*, respectively.

Since the similar expression between ***R****_2_* and ***R****_4_*, we reconstruct a Toeplitz matrix ***R̂***_4_*_T_* from ***R****_4_* in the minimum metric distance sense by solving the following optimization problem [13]:

(13) $$\underset{R_{4T}\in S_{T}}{\min}\left\|R_{4T}-R_{4}\right\|$$

where ***S****_T_* is the set of Toeplitz matrices. The TAM of [14] demonstrates that the optimal approximating Toeplitz matrix ***R̂***_4_*_T_* has the basic entries given below

(14) $${\hat{z}}_{h}={\left(2N-1-h+1\right)}^{-1}\sum_{p=1}^{2N-1-h+1}{\hat{r}}_{p\;(p+h-1)}$$

where the element *r̂_p_*_(_*_p_*_+_*_h_*_−1)_ is the *p*th row and (*p* + *h* − 1)th column of ***R̂***_4_, *h* ∈ [1, 2*N* − 1]. And then ***R̂***_4_*_T_* can be achieved by means of the Toeplitization operator given by

(15) $${\hat{R}}_{4T}=\text{Toep}({\hat{z}}_{1},\ldots \ldots {\hat{z}}_{2N-1})$$

where *Toep* denotes the Toeplitization operator. We then estimate the bearings of signal sources based on the TFOC-MUSIC algorithm using ***R̂***_4_*_T_* which makes the TFOC-MUSIC algorithm more competent for DOA estimation than the MFOC-MUSIC algorithm with ***R̂***_4_.

The procedure of the TFOC-MUSIC algorithm is detailed as follows:

Step 1 Estimate ***Ĉ***_4_ from the received data by array measurements ***X***(*t*) with Equations (5) and (6).
Step 2 Take out the *1*th to *N*th and all *kN*th *(k* = 2, …, *N)* rows of ***Ĉ***_4_ in order, and then store these rows in the *1*th to *2N-1*th row of the ***R̂***_4_ matrix.
Step 3 Gain the columns of ***R̂***_4_ via using its conjugate symmetry for reducing computation.
Step 4 Apply Toeplitz approximation to ***R̂***_4_ for ***R̂***_4_*_T_* as Equations (14) and (15).
Step 5 Remove the redundant items of the expanded steering vector ***b***(*θ*), the rest items can be rewritten as a new vector ***d***(*θ*) = [*1*, …, *p^2N^*^−^*^2^*]*^T^* according to the ascending order.
Step 6 The estimate of DOAs of source directions can be attained by searching the peaks of redefined spatial spectrum *p*(*θ*), which can be expressed as
  (16) $$\begin{array}{cc} P(\theta )=\frac{1}{d{\left(\theta \right)}^{H}U_{N}{U_{N}}^{H}d(\theta )} & \theta \in \left[-90{}^{\circ},90{}^{\circ}\right] \end{array}$$

### 3.3. Complexity

According to the principle of the TFOC-MUSIC algorithm, when compared with the MFOC-MUSIC algorithm, it incurs 2(2*N* − 1) − 1 average operations, 2(2*N* − 1)^2^ − (2*N* − 1) additive operations and 2(2*N* − 1) − 1 conjugate operations. However, the TFOC-MUSIC algorithm can estimate the DOA of more targets with less sensors, it can be considered that the calculation of the TFOC-MUSIC algorithm is approximately in agreement with that of the MFOC-MUSIC algorithm.

## 4. Performance Analysis

In this part, we evaluate the performance of the TFOC-MUSIC algorithm with several experiments in spatially-white noise and in spatially-color noise environment, respectively. The FOC-MUSIC, MFOC-MUSIC and TFOC-MUSIC algorithms are compared in terms of spatial spectrum, normalized probability of success, average maximum estimate deviation and average estimate variance of incoming signals with respect to variables such as angle *θ*, signal-to-noise ratio (SNR) and sampling snapshots *L*. We defined three criteria, namely normalized probability of success (NPC), average maximum estimate deviation (AMED) and average estimate variance (AEV), to evaluate the performance. Define the event that satisfies max (|*θ̂_i_*−*θi*|) < *ε*, *i* = 1,⋯*M* as “success”. Where *ε* equals 0.8 and 1.8 for comparison *versus* SNR and snapshot, respectively. The normalized probability of success equals the times of successes as follows:

(17) $$\text{NPC}=\frac{\text{times of}\;“\text{success}”\;\text{happens}}{\text{MC}}$$

where MC denotes the times of Monte-Carlo simulation. Besides, AMED and AEV are defined as, respectively:

(18) $$\text{AMED}=\underset{\text{MC}}{\text{Ave}}\left[\max\left({\hat{\theta }}_{i}-\theta_{i}\right)\right]=\frac{\sum_{j=1}^{\text{MC}}\max\left({\hat{\theta }}_{i}-\theta_{i}\right)}{\text{MC}},i=1,\cdots M$$

(19) $$\text{AEV}=\frac{\sum_{i=1}^{M}\mathrm{var}{\hat{\theta }}_{i}}{M}=\frac{\sum_{j=1}^{M}\left\{E{\left({\hat{\theta }}_{i}\right)}^{2}-{\left[E\left({\hat{\theta }}_{i}\right)\right]}^{2}\right\}}{M},i=1,\cdots M$$

where *M* is the number of sources. *θ̂* and *θ_i_* represents the estimated and real DOAs, respectively. Consider an isotropic three-element ULA (*N* = 3) with half-wavelength element separation illuminated by three mutually independent far-field signals (*M* = 3) from {−45°, 15°, 40°}. The signals have the non-Gaussian form of *s_l_*(*t*) = *A_l_e^jωlt^*, which are assumed to be of equal power. For convenience, let *A_l_* = 1 for all *l*.

### Case 1

Spatial Spectrum *versus* Angle *θ*

**Figure 1.**
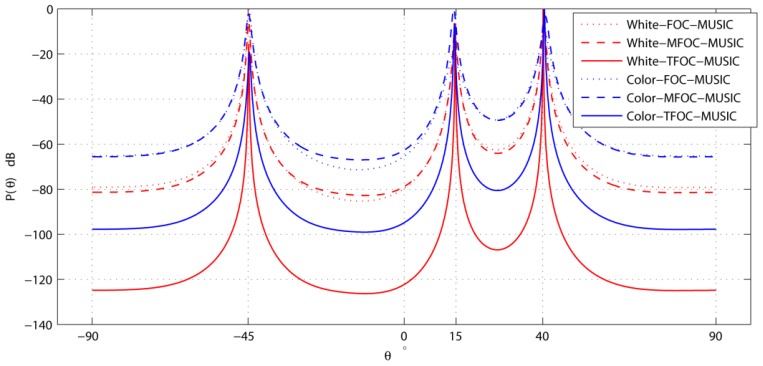
Spatial spectrum comparisons *versus* angle.

Figure 1 exhibits the use of the FOC-MUSIC, MFOC-MUSIC and TFOC-MUSIC algorithms to detect the bearings of three impinging sources in both spatially-white noise and spatially-color noise situations. Here, the SNR at each sensor is 10 dB, and *L* = 1,000. As depicted in Figure 1, the three sources have been successfully detected in above-mentioned two types of noise by the three algorithms. However, the angular resolution of the TFOC-MUSIC algorithm is much higher than the FOC-MUSIC and MFOC-MUSIC algorithms. The reason for the improvement of angular resolution with the TFOC-MUSIC algorithm is that ***R̂***_4_*_T_* achieved with Toeplitz approximate method is further close to the desired ***R*_4_** than ***R̂***_4_ in the same condition.

### Case 2

Normalized Probability of Success, Average Maximum Estimate Deviation and Average Estimate Variance *versus* SNR

**Figure 2.**
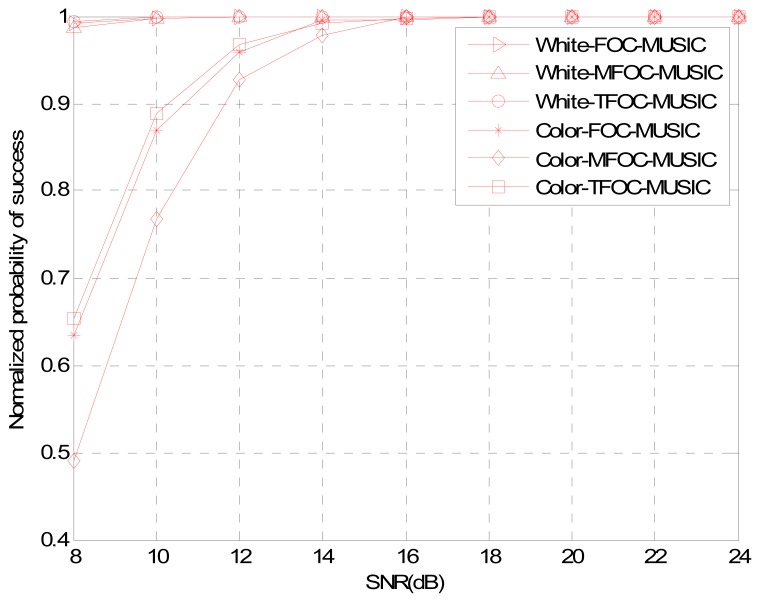
Normalized probability of success comparisons *versus* SNR.

**Figure 3.**
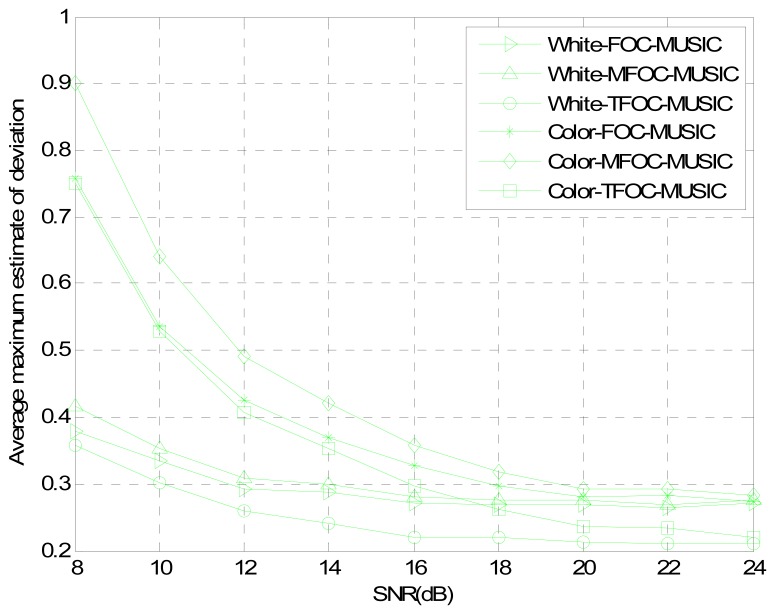
Average maximum estimate deviation comparisons *versus* SNR.

**Figure 4.**
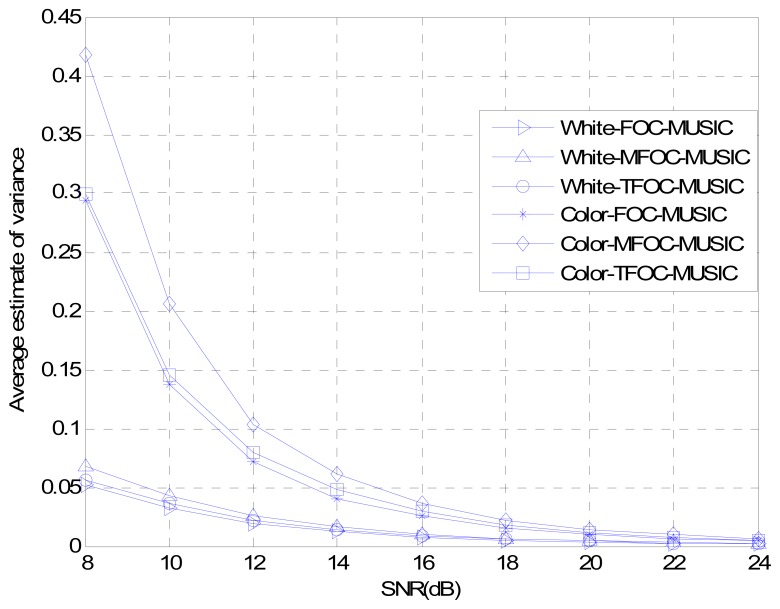
Average estimate variance comparisons *versus* SNR.

The estimated performances of normalized probability of success, average maximum estimate deviation and average estimate variance are plotted in Figures 2–4 as a function of the SNR, respectively. Snapshots *L* are set to be 2,000, and 200 Monte Carlo's runs are carried out for estimators. As can be seen from three pictures, it is obvious that for low SNRs, the TFOC-MUSIC algorithm outperforms the FOC-MUSIC and MFOC-MUSIC algorithms in all the three performances metrics. Moreover, as the SNR increases, the performance curves of each figure tend to become consistent by and large. But the TFOC-MUSIC algorithm decreases the complexity by removing the matrix redundancy. The better behavior of the TFOC-MUSIC algorithm is also determined by recovering the Toeplitz structure of ***R̂***_4_ using Toeplitz approximation which improves the performance of DOA estimation.

### Case 3

Normalized Probability of Success, Average Maximum Estimate Deviation and Average Estimate Variance *versus* Snapshots *L*

**Figure 5.**
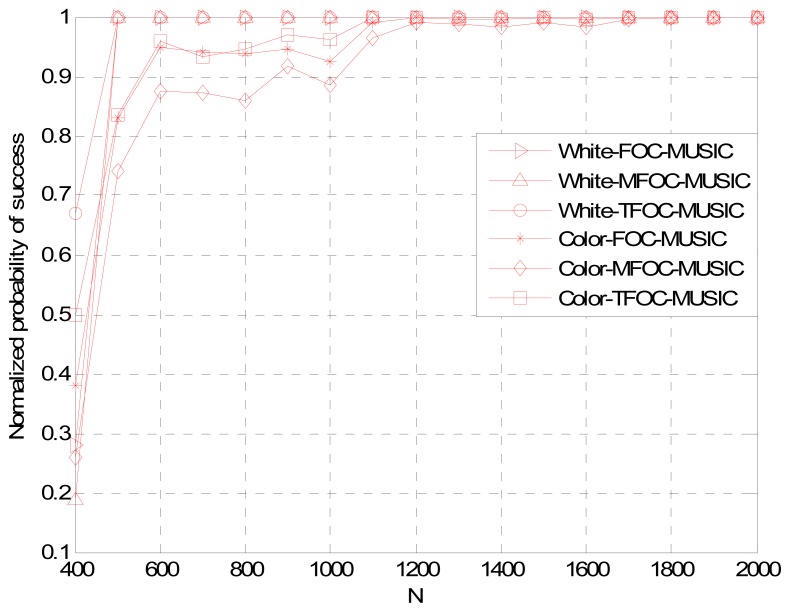
Normalized probability of success comparisons *versus* snapshots *L*.

**Figure 6.**
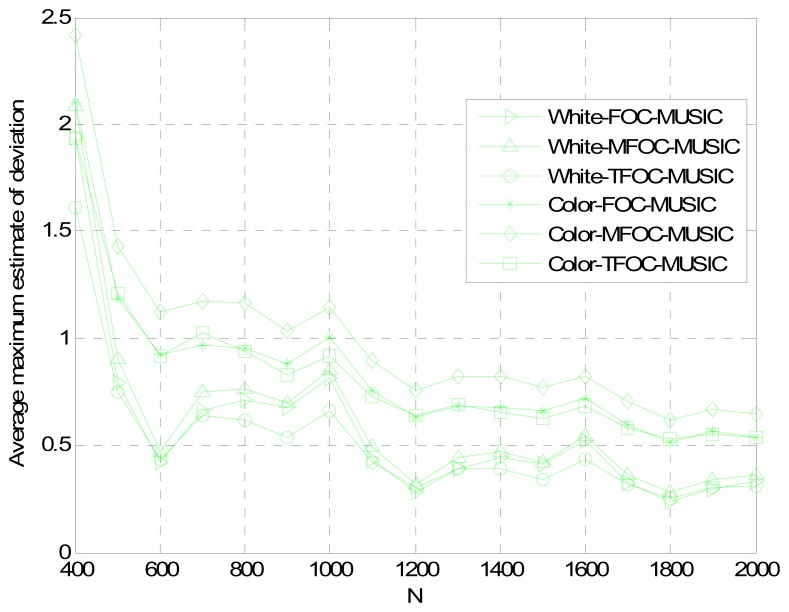
Average maximum estimate deviation comparisons *versus* snapshots *L*.

**Figure 7.**
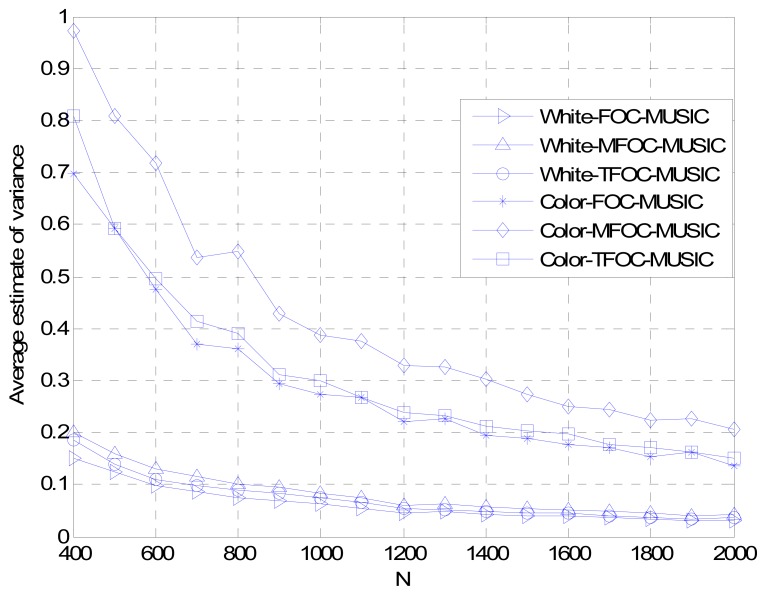
Average estimate variance comparisons *versus* snapshots *L*.

Figures 5–7 show the DOA estimation performance with the FOC-MUSIC, MFOC-MUSIC and TFOC-MUSIC algorithms in the same setting with SNR = 10 dB, 500 Monte Carlo's simulations setup. From the simulation results (Figures 5–7), it is clearly indicated that the curves obtained by the TFOC-MUSIC algorithm are much better than those by the FOC-MUSIC and the MFOC-MUSIC algorithms either in spatially-white or spatially-color noise situation. And the three pictures' curves display sharp fluctuation for snapshots from 400 to 600 due to the small snapshots case, the estimate matrix ***R̂***_4_ deviates from the ideal ***R*_4_** much further.

In addition, the performance curves become stabilized with an increasing data length. Hence, we ascertain that the estimated performance becomes optimal, since the snapshots number goes to infinity. But in the convergence progress, the complexity of the TFOC-MUSIC algorithm is obviously smaller than that of the FOC-MUSIC algorithm, and the convergence speed of the TFOC-MUSIC algorithm is much faster than that of the MFOC-MUSIC algorithm. Complexity reducing benefit from the lower cumulants matrix rank dimension of the TFOC-MUSIC algorithm compared to the FOC-MUSIC algorithm. The cumulants matrix reconstructed using the Toeplitz approximate method is close to the desired ***R***_4_ than the MFOC-MUSIC algorithm in the same condition, which helps speed up the convergence.

To sum up, as can be noticed from Figures 1 to 7, compared with the MFOC-MUSIC algorithm, the TFOC-MUSIC algorithm behaves better in spatial spectrum estimation, normalized probability of success, average maximum estimate deviation and average estimate variance of incoming signals for both spatially-white noise and spatially-color noise situations. The modified Toeplitz structure of reduced-rank ***R̂***_4_, namely, ***R̂***_4_*_T_* contributes to the improvement of the performance of DOA estimation while yet maintaining property extending the effective array aperture of a physical array. Besides, the complexity increase is not obvious for a small array size.

## 5. Conclusions

A novel DOA estimation algorithm has been presented in this paper. Its main idea is to utilize the MFOC-MUSIC algorithm in conjunction with Toeplitz approximation. In this way, the effective array aperture of a physical array can be extended that allows the number of estimated signals to be greater than or equal to that of sensors. Moreover, for non-Gaussian sources, in contrast to the MFOC-MUSIC algorithm, the proposed method has lower average maximum estimate deviation and average estimate variance, higher normalized probability of success and angular resolution. And the threshold of snapshots is less than that of the MFOC-MUSIC algorithm to some extent. In addition, the computation of the TFOC-MUSIC algorithm is approximately consistent with that of the MFOC-MUSIC algorithm, while obviously smaller than the FOC-MUSIC algorithm due to estimating DOA of more targets with less sensors. Simulation results show that the proposed method is more effective and efficient than the MFOC-MUSIC algorithm in DOA estimation, both in spatially-white noise and spatially-color noise situations.
